# A technique for optimal manipulation of rotation of the flexible ureterorenoscope

**DOI:** 10.1308/003588412X13373405385214j

**Published:** 2012-07

**Authors:** G Ellis, S Pridgeon, S Graham

**Affiliations:** Whipps Cross University Hospital NHS TrustUK

## BACKGROUND

Three different types of movement are required to perform flexible ureterorenoscopy: insertion/retraction, rotation and deflection of the tip. Many trainee urologists struggle to manipulate the rotation of the scope. We describe a technique for optimally controlling this rotation.

## TECHNIQUE

**Figure 1:**
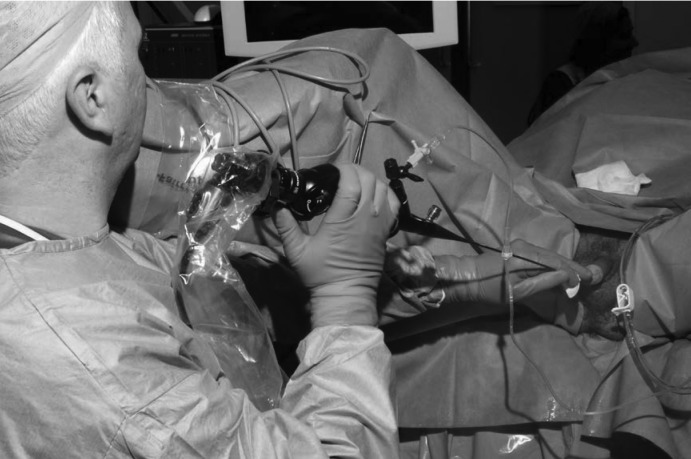
Holding the flexible ureterorenoscope extended in a straight line, gentle traction is applied at opposing ends of the scope in a ‘Christmas cracker’ motion.

**Figure 2:**
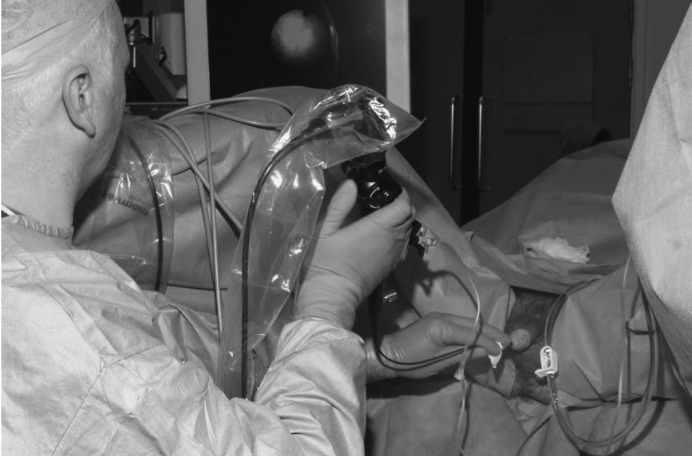
Holding the flexible ureterorenoscope in a curved position

When performing ureterorenoscopy, the scope is extended in a straight line (Fig 1) rather than held in a curved position (Fig 2). By applying gentle opposing traction with each hand in a ‘Christmas cracker’ motion this can be optimally achieved.

## DISCUSSION

Many surgeons default to holding the scope in a curved position. Due to the length and the ever decreasing calibre of modern scopes, the transmission of torque is reduced, resulting in decreased rotation of the tip in response to movements of the hand. Consequently, the operator resorts to excessive movements in a ‘windscreen wiper’ motion. Using this technique improves the ability of the surgeon to control the rotation of the scope and to perform flexible ureterorenoscopy with economy of movement in a safe and efficient fashion.

